# Current Challenges in the Management of Infective Endocarditis

**DOI:** 10.3389/fmed.2021.641243

**Published:** 2021-02-22

**Authors:** Guillermo Cuervo, Francesc Escrihuela-Vidal, Carlota Gudiol, Jordi Carratalà

**Affiliations:** ^1^Infectious Diseases Department, Institut d'Investigació Biomèdica de Bellvitge (IDIBELL), Bellvitge University Hospital, University of Barcelona, Barcelona, Spain; ^2^Spanish Network for Research in Infectious Diseases (REIPI), Instituto de Salud Carlos III, Madrid, Spain; ^3^Insitut Català d'Oncologia, Institut d'Investigació Biomèdica de Bellvitge (IDIBELL), Hospital Duran i Reynals, Barcelona, Spain

**Keywords:** infective endocarditis, transesophageal echocardiography - three-dimensional echocardiography, positron - emission tomography, *Staphycoccus aureus*, viridans group streptococci, *Enterococcus*

## Abstract

Infective endocarditis is a relatively rare, but deadly cause of sepsis, with an overall mortality ranging from 20 to 25% in most series. Although the classic clinical classification into syndromes of acute or subacute endocarditis have not completely lost their usefulness, current clinical forms have changed according to the profound epidemiological changes observed in developed countries. In this review, we aim to address the changing epidemiology of endocarditis, several recent advances in the understanding of the pathophysiology of endocarditis and endocarditis-triggered sepsis, new useful diagnostic tools as well as current concepts in the medical and surgical management of this disease. Given its complexity, the management of infective endocarditis requires the close collaboration of multidisciplinary endocarditis teams that must decide on the diagnostic approach; the appropriate initial treatment in the critical phase; the detection of patients needing surgery and the timing of this intervention; and finally the accurate selection of patients for out-of-hospital treatment, either at home hospitalization or with oral antibiotic treatment.

## Introduction

First described by the French physician Lazare Rivière more than 350 years ago, the clinicopathological manifestations of the infection of heart valves were better characterized through the enormous contributions of William Osler at the end of the 19th century (1). The infection affects the endocardial surface of the heart, most commonly the valves, but also may occur on mural endocardium, on cardiac septal defects, on arteriovenous or arterioarterial shunts and on intravascular devices. The current name of this infection, infective endocarditis, was popularized in the 1960s by Lerner and Weinstein to cover other possible, but infrequent etiologies in addition to bacterial infections (2).

Although the incidence of infective endocarditis seems to be slightly increasing (3), this disease continues to be a relatively rare, but severe cause of sepsis. Currently, up to 40–50% of affected patients require valve surgery at some point during the clinical course, with overall mortality remaining around 20–25% per year in most published series (4). Although the classic clinical syndromes of acute or subacute endocarditis are still observed to a certain degree, current clinical forms have changed. Furthermore, there have been profound epidemiological changes in high-income countries, with a clear and progressive increase in the number of cases associated with prosthetic valves and intravascular devices (3).

In this narrative review, we will address: (1) the epidemiological changes mentioned above; (2) several new advances in the understanding of the pathophysiology of infective endocarditis and in endocarditis-triggered sepsis; (3) new diagnostic tools; and finally (4) therapeutic aspects such as the relevance of early surgical treatment in selected cases, new available drugs and new useful treatment strategies.

The PubMed database was used to search medical literature published in English from 1st January 2010 to 30th October 2020, using the search terms “infective endocarditis” AND “epidemiology” OR “pathophysiology” OR “diagnosis” OR “treatment” OR “management.” We reviewed both original and review articles, excluding case reports and editorial articles. Some earlier published articles were also included due to their relevance to this review.

## Epidemiology

Infective endocarditis is considered an infrequent disease, with an annual incidence ranging from 1.5 to 15 cases per 100,000 inhabitants. It displays significant international variation (5, 6). The highest rates have been found in the United States, while the incidence is lower in Denmark. There is a significant lack of epidemiological information from Asia, Oceania and Latin America. Furthermore, the incidences can vary significantly even within the same country (7). Even though the scarcity of epidemiological data, particularly from low-income countries, a slight increase in the incidence of infective endocarditis has been noted since 2000. In this regard, controversy remains about the true impact of restricting antibiotic prophylaxis in high-risk patients, as recommended by NICE guidelines (8), with some investigations showing an increasing trend of infective endocarditis afterwards. The efficacy and impact of this strategy on antibiotic resistance are yet to be fully addressed (9).

### Toward a Nosocomial Profile of Infective Endocarditis

A predisposing condition, such as rheumatic heart disease, is nowadays less commonly detected among cases of infective endocarditis, although the importance of such predisposing conditions persists in low-income countries (10). Cases associated with intravenous drug use have decreased globally, but a dramatic increase of this habit continues to affect the epidemiology of endocarditis in North America (11) and in some Eastern European countries (12). Other risk factors are being increasingly detected in high-income countries, such as degenerative valve disease, intracardiac devices (both cardiovascular implantable electronic devices as well as left ventricular assist devices), indwelling catheters and immunosuppression. This explains why the latest analyses of the demographics of endocarditis cases show a trend toward nosocomial characteristics in high-income countries: older patients, staphylococcal (both *Staphylococcus aureus* and coagulase-negative staphylococci) (13, 14) and enterococcal cases (15), and the involvement of prosthetic valves and cardiovascular implantable electronic devices (CIEDs) (3, 4, 11, 16, 17). By contrast, the oral streptococcal (so-called “subacute”) endocarditis classically associated with rheumatic heart disease has become less frequent (5, 18).

### Infrequent Etiologies and Culture-Negative Endocarditis

Other etiologies of endocarditis are infrequent: 2–5% of cases can be produced by Gram-negative bacilli (both aerobic Gram-negative bacilli or by the known HACEK group: *Haemophilus* spp., *Aggregatibacter actinomycetemcomitans, Cardiobacterium hominis, Eikenella corrodens, Kingella kingae*). Also uncommon are fungal endocarditis that can represent <2% of cases, mostly produced by yeasts of the *Candida* spp. genus or rarely by other yeasts or filamentous fungi.

Finally, a variable proportion (up to 10–20% of cases) without documented etiology are considered “culture-negative endocarditis,” mostly as a consequence of prior administration of antibiotics or caused either by fastidious slow-growing microorganisms or by truly non-cultivable intracellular bacteria (e.g., *Coxiella burnetii, Chlamydophila* spp, *Bartonella* spp, *Tropheryma whippelii*) (4, 19).

### Infective Endocarditis Associated With Transcatheter Aortic Valve Implantation

Transcatheter aortic valve implantation (TAVI) has led to a revolution in the management of valvular heart disease. This technique has become a suitable alternative to surgery in elderly patients with aortic valve stenosis who carry a high or moderate surgical risk (20). Therefore, the number of TAVI procedures has dramatically increased in recent years (21). The incidence of endocarditis associated with TAVI has been estimated to be 0.8–1.4% (22, 23). A meta-analysis comparing endocarditis following TAVI vs. surgical replacement found no difference in the overall incidence (24). Endocarditis after TAVI displays characteristics of healthcare-associated infections, with a high predominance of staphylococcal and enterococcal infections (23, 25, 26). In-hospital mortality of TAVI-associated endocarditis is elevated, strongly influenced by the epidemiological profile of the patients (27).

### Endocarditis and Sepsis

Sepsis and septic shock are severe complications that may arise from any type of infection, with poor early and late prognoses in the patients affected. A study on 894 episodes of infective endocarditis showed that 17.4% of the patients had septic shock at any time during hospitalization (28). A multivariate analysis suggested that *S. aureus* and signs of a persistent infection were independent predictors for the development of septic shock, alongside a previous diagnosis of diabetes mellitus and other systemic complications such as acute kidney injury and supraventricular tachycardia. Furthermore, the multivariate study indicated that the development of septic shock at any time during hospitalization was strongly associated with in-hospital mortality. Similar results were obtained in a study comparing endocarditis diagnosed “early” or “late” after the development of first symptoms. That study showed that the so-called “acute endocarditis” was more frequently presented as or complicated with septic shock (29). Sepsis and septic shock are associated with a 4-fold increase in the probability of death (28, 30).

### Morbidity and Mortality

Despite advances in diagnosis and therapeutics, infective endocarditis presents a significant morbidity burden and a remarkably high overall mortality (20–25% of cases). Endocarditis-related mortality has remained steadily high since 2000 and is strongly associated with several risk factors, such as advanced age, a high Charlson comorbidity index, non-community acquisition, prosthetic valves, staphylococcal infections, perivalvular complications, stroke, and the non-performance of surgery when indicated (3, 4, 11, 16, 31, 32). Of note, studies on psychological outcomes in survivors after an episode of endocarditis have demonstrated a reduction in quality of life and the occurrence of posttraumatic stress disorder (33).

## Infective Endocarditis Pathophysiology

The concurrence of several pathogenic events is required for the development of infective endocarditis, which partly explains the relatively low incidence of this disease.

From the seminal studies performed in animal models, it has been well known that inducing infective endocarditis in the absence of pre-existing endothelial damage is extremely difficult (34, 35). Important predisposing conditions are prior valvular involvement, classically rheumatic or currently due to degenerative disease (as well as the presence of prostheses or endovascular devices). These structural alterations induce turbulent blood flow that causes mechanical stress on the vascular wall, ultimately producing endothelial injury. More recently, the ability to induce experimental endocarditis in structurally healthy, but inflamed valves has been demonstrated (36). This mechanism could explain the development of endocarditis in previously normal hearts in patients with infections caused by aggressive microorganisms (e.g., *S. aureus*) and with endothelial inflammation caused by sepsis itself or by other agents that cause vascular damage.

After the initial endothelial damage or inflammation, the second key pathogenic event is the deposition of sterile fibrin-platelet aggregates in these injured areas. These lesions, leading to what is known as “non-bacterial thrombotic endocarditis,” have been described in up to 2.4% of the autopsies performed in patients with certain underlying diseases (37) and are the ideal niche for the subsequent anchoring of the bacteria seeded in the bloodstream. Not all bacteria from the bloodstream, however, have the same ability to colonize these lesions (38). Gram-negative bacilli, for example, are particularly susceptible to humoral innate immune responses (39). Furthermore, certain Gram-positive microorganisms, particularly some species of streptococci and *S. aureus*, have specific molecules on their surfaces called adhesins, such as “microbial surface components recognizing adhesive matrix molecules” (MSCRAMMs) and “secretable expanded repertoire adhesive molecules” (SERAMs). These adhesins recognize integrins, specific ligands located on the injured or inflamed endothelial surface. Since the 1980s to 1990s, a repertoire of molecules of the MSCRAMM and SERAM type have been described in detail for streptococci and *S. aureus* (40–44), which interact not only with the endothelium, but also with platelets and key proteins of the clotting cascade (45–47).

The last important pathogenic event is the maturation and growth of the fibrin-platelet aggregates, which clump together with bacteria at a high inoculum (more than 10^9^ colony-forming units per gram of vegetation) in what is known as “vegetation.” Within this vegetation, bacterial communities are partly organized into complex biofilms, embedded in the fibrin-platelet aggregate and in a matrix of macromolecules produced by them. The arrival of phagocytes and antibiotics can be compromised in these structures, inside which bacteria can also modify their metabolism toward persistent phenotypes, with greater tolerance to antibiotics (48–50). The vegetation represents the pathological hallmark of infective endocarditis and determines its main clinical manifestations, namely: (1) the growth of this “full bacterial lesion” causes continuous bacteremia at a high inoculum that can seed distant septic metastases, (2) the invasion of the structures to which this vegetation is anchored can cause valvular destruction, negatively affecting the patient's hemodynamics, and finally (3) this friable mass can detach pieces that cause distant embolisms, which can significantly affect the function and prognosis of the affected patients. In recent years, intensive studies have been carried out in this field. Through pharmacological manipulation, successful attempts have been made to hinder the interactions between bacteria and the endothelium, effectively preventing the development or modulating the severity of infective endocarditis in animal models treated with antiplatelet and anticoagulant drugs (51, 52). Although the possible prophylactic role of some antiplatelet or anticoagulant drugs has been reinforced by observational clinical studies (53–55), the prophylactic use of these drugs has not been effectively transferred to clinical practice yet (56).

### Endocarditis-Triggered Sepsis and Septic Shock

As described previously, a non-negligible proportion of patients with endocarditis may present with severe sepsis or septic shock (28), which can eventually lead to multi-organ failure. This complication appears to be associated with some characteristics of the patients (57) as well as with particularly virulent microorganisms such as *S. aureus* and beta-hemolytic streptococci (58). In addition to their invasive and destructive effects on the affected anatomical structures, these bacteria can seed distant septic metastases. Moreover, they display a repertoire of other virulence mechanisms, including the excretion of exotoxins that can act as superantigens, which overactivate the immune system (59–61). The systemic inflammation that is consequently triggered has an important hemodynamic impact, with generalized endothelial dysfunction and a drop in vascular resistance. Any increases in the compensatory cardiac output may be hampered by sepsis itself, a phenomenon known as “septic cardiomyopathy” (62), or by the destruction of the valve as a result of the infection. This extremely serious situation explains why the presence of septic shock is associated with a significant increase in the risk of mortality.

## Diagnostic Tools

The diagnosis of infective endocarditis relies on a combination of clinical, microbiological and imaging information, as specified by the modified Duke criteria (63, 64). The classic combination of clinical features of infective endocarditis remains a critical feature in diagnosis, particularly for subacute or chronic endocarditis. The mainstay of diagnosis involves the information provided by blood cultures and different imaging techniques that can detect anatomical changes such as valve vegetations or associated complications. However, a shift toward more acute infections and the involvement of prosthetic materials have decreased, to some extent, the usefulness of applying these classic clinical features in diagnosis. The performance of the modified Duke criteria has been thus compromised in the era of non-community acquired infective endocarditis. Therefore, modifications have been proposed to improve their sensitivity (65, 66). Nowadays, the diagnostic accuracy of these criteria seems to largely rely on the development of new and more sensitive non-invasive imaging techniques (Table 1).

**Table 1:** Imaging and laboratory techniques used in the diagnosis of infective endocarditis.

Transthoracic echocardiography (TTE)	Mainstay of diagnosis. Limited sensitivity in prosthetic valve endocarditis and paravalvular complications.
Transesophageal echocardiography (TOE)	Mainstay of diagnosis. Indicated in prosthetic valve endocarditis and for the detection and characterization of valvular and paravalvular complications.
Three-dimensional transesophageal echocardiography (3D-TOE)	Complement to TOE when characterizing valvular and paravalvular complications. Surgical planning
Intraoperative transesophageal echocardiography	Routine exploration when surgery is performed. Assessment of post-operative anatomy and ventricular function.
Cardiac computed tomography (CT)	Better characterization of complications involving an aortomitral fibrous body and the aortic root. Alternative to coronary angiography for exploration in patients with aortic endocarditis.
Positron emission tomography/computed tomography (PET/CT)	Major Duke criteria for diagnosis of prosthetic valve endocarditis and device-associated endocarditis. Increasing use in infective endocarditis associated with TAVI and LVAD. Detection of extracardiac complications of endocarditis or alternative sources of infection.
Radiolabeled white blood cell single-photon emission computed tomography/computed tomography (WBC-SPECT/CT)	Complement to PET/CT when results are non-conclusive. Similar performance to that of PET/CT.
Brain magnetic resonance imaging (MRI)	Detection and characterization of silent or clinically evident intracranial complications. Evaluation of evolving changes in intracranial complications that may influence the timing for surgery.
Thoracoabdominopelvic computed tomography	Not recommended as a routine exploration in asymptomatic patients.
Contrast-enhanced ultrasound	Promising results on detecting intraabdominal emboli as an alternative to CT.
Colonoscopy	Detection of colorectal disease or neoplastic processes in *Streptococcus gallolyticus* or *Enterococcus faecalis* endocarditis.
Culture and polymerase chain reaction of samples from explanted heart valves	Improves the diagnostic performance of the Duke criteria.
Serum cytokine profiles and bacteria-targeting tracers	Under research; not routinely used.

*TAVI, transcatheter aortic valve implantation; LVAD, left-ventricle assistance devices*.

### Echocardiography

Transthoracic echocardiography (TTE) is the main imaging method in the diagnosis of endocarditis, with varying sensitivity rates for valvular and paravalvular abnormalities such as vegetations (sensitivity around 65%), a new regurgitation or dehiscence of a prosthetic valve, perforations, abscesses and fistulae (64). Transesophageal echocardiography (TOE) provides a better detection and characterization of local abnormalities (sensitivity for intracardiac vegetations of ~95%), particularly when TTE is negative, in the case of valvular or paravalvular complications as well as in prosthetic valve endocarditis (PVE) and endocarditis associated with CIEDs (67).

Three-dimensional TOE (3D-TOE) may complement conventional TOE. Although its contribution to the diagnosis of endocarditis is not clearly established (68, 69), its main value is in providing a detailed description of vegetations, regurgitations and abscesses in both native (70) and prosthetic valve endocarditis (71). This technique can also differentiate vegetations from thrombi (72) and can be used in surgical planning. Intraoperative TOE has been demonstrated to be useful in the surgeries for endocarditis and has been proposed for use in routine exploration (73, 74).

### Cardiac Computed Tomography

Although TOE remains the mainstay in the diagnosis of infective endocarditis, there is growing interest in the application of cardiac computed tomography (CT). This tool shows good anatomical correlation, especially when diagnosing a perivalvular abscess of the aortomitral intervalvular fibrous body and other structures surrounding the aortic root, thus overcoming the limitations of TOE (75–77). Recent data support cardiac CT as an adjuvant exploration technique when a better depiction of valvular complications is needed or when echocardiography proves to be insufficient in both native (77) and prosthetic valve endocarditis (78–80). Furthermore, cardiac CT is frequently used to preoperatively assess the presence of coronary artery disease in aortic endocarditis where performing a coronary angiography carries a prohibitive high risk of the dislodgment of vegetations (74). A recent study from Wang and colleagues added a prognostic value to cardiac CT, suggesting a synergistic role with TOE in surgery planning and in predicting early and late mortality (81).

### Positron Emission Tomography/Computed Tomography (PET/CT)

To complement the detection of anatomic abnormalities, progress has been made in measuring biological activity through ^18^F-fluorodeoxyglucose positron emission/computed tomography (FDG-PET/CT) and radiolabeled white blood cell single-photon emission CT/CT (WBC-SPECT/CT). Their use has been recommended by the European Society of Cardiology in patients with suspected PVE for valves implanted for more than 3 months (74), with a positive result included as a major criterion for the diagnosis of prosthetic valve and device-related endocarditis. Prospective studies of patients with suspected PVE have revealed a remarkable performance of FDG-PET/CT and WBC-SPECT/CT in the diagnosis of PVE (82, 83). They suggest that these two imaging techniques can be used in a stepwise fashion when evaluating the presence of endocarditis. FDG-PET/CT should be used first, since it has higher sensitivity, and when the results are not conclusive, WBC-SPECT/CT may be performed. Similar results have been obtained with both FDG-PET/CT (84, 85) and WBC-SPECT/CT (86–89) for suspected CIEDs endocarditis. Controversy remains on the use of FDG-PET/CT in patients with aortic root grafts with a prosthetic valve, since a high rate of false positives has been observed in relation to surgical adhesives (82, 90). FDG-PET/CT performs very well in the diagnosis of PVE when adjusting for confounders such as the low inflammatory activity caused by the initiation of antibiotic treatment (82, 90, 91), suggesting that FDG-PET/CT should be performed as soon as possible when infective endocarditis is suspected. Furthermore, FDG-PET/CT has proven prognostic value in PVE by correlating with major cardiac events (92).

The role of FDG-PET/CT in the diagnosis of native valve endocarditis has not been fully established and may be limited to cases where endocarditis is strongly suspected but the Duke criteria are not totally met. Studies on the use of FDG-PET/CT in native valve endocarditis are mostly retrospective and might overestimate the sensitivity of this technique (93, 94). In such cases, FDG-PET/CT may have an impact on diagnosis by detecting extracardiac complications of endocarditis (95).

The increasing use of TAVI and left ventricular assist devices (LVAD) have led to challenges in diagnosing the infective complications associated with their use. A recent study in patients with suspected TAVI-related endocarditis showed that the inclusion of FDG-PET/CT led to the reclassification of 36% of the patients diagnosed with “possible endocarditis” by the modified Duke criteria (96). Moreover, FGD-PET/CT and WBC-SPECT/CT have been used in cases of endocarditis associated with LVADs, showing variable rates of sensitivity and specificity (97–100). WBC-SPECT/CT and FDG-PET/CT show similar sensitivity and specificity, but the former may be more challenging to perform since it uses a more difficult protocol and requires the manipulation of blood specimens.

FDG-PET/CT is also useful in revealing unexpected sources of primary infections and detecting extracardiac complications of endocarditis. Thus, its use can lead to changes in treatment plans (101). However, no recommendations have been made in international guidelines regarding the detection of extracardiac complications.

### Cerebral Imaging

Recommendations for neuroimaging in infective endocarditis remain unclear. Brain CT is often used when neurological symptoms are present, although brain magnetic resonance imaging (MRI) has better sensitivity in defining lesions. Clinically silent complications of the central nervous system, such as embolisms, may occur in up to 60% of patients (102). Some centers routinely perform brain MRI when infective endocarditis is diagnosed since it may provide additional diagnostic findings for fulfilling the modified Duke criteria and may change therapeutic plans (103) or the timing for surgery (104). However, while major intracranial hemorrhages and extensive ischemic stroke worsen prognosis after valve surgery, brain MRI findings of clinically silent complications do not affect postoperative mortality (105). In this sense, the clinical significance of cerebral microbleeds, one of the most frequently encountered silent lesions, remains to be elucidated (106, 107).

### Other Diagnostic Tools

Systematic thoracoabdominopelvic CT has not demonstrated a clear utility in the diagnosis of left-sided endocarditis in asymptomatic patients. Furthermore, it increases the risk of kidney toxicity (108). Nevertheless, the finding of pulmonary embolisms in chest CT may be useful in the diagnostic workup of right-sided endocarditis either on the tricuspid native valve in intravenous drug addicts or associated with pacemakers (109). Contrast-enhanced ultrasound may be useful as an alternative for the detection of abdominal complications, mostly spleen infarctions (110).

Regarding the detection of the portals of bacterial entry, colonoscopy has proved to be very useful. The relationship between *Streptococcus gallolyticus* and colon cancer has been well known since the 1950s (111). Two observational studies on infective endocarditis caused by *Enterococcus faecalis* found a high rate of colorectal disease when a colonoscopy was performed, with a high incidence of neoplastic disease particularly in those with an unknown source of bacteremia (112, 113). A systematic and multidisciplinary search for portals of bacterial entry has been proposed, suggesting that a meticulous physical examination should be performed when evaluating patients with infective endocarditis (114).

Procedures involving polymerase chain reaction (PCR) using samples from explanted heart valves could improve diagnostic performance (115) in comparison with conventional cultures. Although interesting from a theoretical point of view, those molecular techniques applied to the detection of genetic material in blood samples for cases of culture-negative endocarditis have shown low sensitivity so far (116). Finally, there is an increasing interest in finding new predictors of mortality in patients with endocarditis through the use of serum cytokine profiles (117) or bacteria-targeting tracers alongside diverse nuclear imaging techniques (118). Regarding acute-phase reactants and biomarkers of severe infection, procalcitonin levels are significantly increased in the cases of endocarditis complicated with sepsis or septic shock when compared to cases without these complications (119). No other biomarker has demonstrated a good predictive value in this setting. Current research of biomarkers in endocarditis is focused on proteomic analysis of some molecules that are not yet in routine clinical use (120).

To conclude, progress has been made through the development of new diagnostic techniques and improvements in known ones. However, for a complex and systemic disease such as infective endocarditis, it seems that diagnosis will be further improved by using a refined combination of clinical, microbiological and multimodal imaging information.

## Management

The available evidence to guide antibiotic treatment of infective endocarditis is composed mostly of data from observational studies and some from experimental animal models. A recent Cochrane review analyzed the evidence from six small clinical trials evaluating various antibiotic regimens for endocarditis with a range of etiologies. After an in-depth analysis, the authors of the review concluded that given the high risk of bias, insufficient data, or underpowered designs, the evidence offered by these trials did not support or reject any of the regimens evaluated (121). This poor evidence may partly explain the significant heterogeneity in the management of this disease observed in some surveys (122) and the lack of adherence to some recommendations of the European guidelines even among those who developed them (123).

With these caveats in mind, when treating this infection, the pathophysiological peculiarities of infective endocarditis must be taken into account, such as the presence of very high concentrations of bacteria protected from immune responses within vegetations and the potential existence of bacteria with reduced metabolism embedded in biofilms. For these reasons, it has been postulated that to achieve microbiological eradication, the treatment of infective endocarditis must involve bactericidal antibiotics administered parenterally in high doses and for prolonged periods (124).

The selection of the antibiotic regimen for a particular case of endocarditis is often complex and beyond the scope of this review. All the details on this are available in the published guidelines (74, 125). However, in the next few paragraphs, we will offer a brief overview of the common regimens that are used against the most frequent microorganisms that cause infective endocarditis.

### Antibiotic Therapy

#### Cell Wall-Active Antibiotic Treatment

The cornerstone of the antibiotic treatment of endocarditis is the use of beta-lactams in high doses: penicillin or ceftriaxone for the viridans group streptococci, ampicillin for *E. faecalis*, and antistaphylococcal penicillins or first-generation cephalosporins for methicillin-sensitive *S. aureus* or coagulase-negative staphylococci. In allergic patients or in those with infections caused by strains resistant to beta-lactams, the alternative is usually another cell wall-active agent, such as vancomycin for the viridans group streptococci and *E. faecalis* or vancomycin or daptomycin for staphylococci. Due to the limited amount of evidence, treatment regimens for endocarditis caused by coagulase-negative staphylococci are usually extrapolated from those recommended for treating endocarditis caused by *S. aureus* (126).

#### Antibiotic Combinations

The synergistic combination of aminoglycosides and beta-lactams demonstrates rapid bactericidal action, allowing treatment to be shortened to 2 weeks for native valve endocarditis caused by susceptible viridans group streptococci (127). This antibiotic combination is also recommended for treating infections caused by the viridans group streptococci that are partially or fully resistant to penicillin, although this recommendation is based on less robust evidence (128, 129).

The combination of ampicillin and aminoglycosides is essential for at least the first 2 weeks of treatment (130) to reduce the risk of relapse in endocarditis caused by *E. faecalis*, given the relative tolerance of these bacteria to beta-lactams (which is also observed in other species such as *Granulicatella adiacens* and *Abiotrophia defectiva*). When this combination cannot be used due to high-level aminoglycoside resistance or an unacceptable risk of toxicity, combination with ceftriaxone (so-called “double beta-lactam”) is recommended, which achieves bactericidal action presumably through the complementary saturation of penicillin-binding proteins (PBPs) (131, 132).

In endocarditis caused by *S. aureus*, the combination of beta-lactams and aminoglycosides is currently not recommended for native valve infections due to the increased risk of renal toxicity without any relevant clinical benefit (133, 134). It is only recommended for PVE according to experimental data, although some retrospective series question its usefulness even in that scenario (135).

The use of rifampicin in a combination treatment for native valve endocarditis caused by *S. aureus* is discouraged (136), but could be useful in PVE, given its potent activity in infections involving biofilms. Guidelines recommend this regimen for PVE, but only after clearing blood cultures to avoid the emergence of resistant mutants during treatment (136). In any case, this drug may not be essential after valve replacement surgery in patients operated during the active phase of treatment (137).

There is enormous interest in the potential utility of the combination of beta-lactams with daptomycin or fosfomycin in endocarditis caused by methicillin-susceptible *S. aureus*. Experimental evidence shows that combinations of both drugs with cloxacillin produce greater sterilization of vegetations on the left-sided valves in animal models (138). However, this potential beneficial effect of the combination with daptomycin was not observed in a retrospective study conducted in our center (139) or in a recent clinical trial of bacteremia caused by susceptible *S. aureus* (140), although it is worth mentioning that there was an underrepresentation of infective endocarditis as a cause of bacteremia in both studies (9 and 10% of patients, respectively). The combination of cloxacillin with fosfomycin in bacteremia and endocarditis caused by susceptible *S. aureus* is an attractive alternative. In an ongoing multicenter clinical trial (SAFO trial) we are testing this combination for bacteremia caused by methicillin-susceptible *S. aureus* (141).

In the case of endocarditis caused by methicillin-resistant *S. aureus* (MRSA), combination therapy is likely to be more effective given the relatively poor results of both vancomycin and daptomycin monotherapy. The possible synergistic effect of vancomycin and beta-lactams in MRSA bacteremia suggested by *in vitro* studies, retrospective studies (142, 143) and a pilot clinical trial (144) could not be confirmed in the international multi-center CAMERA 2 clinical trial. This trial had to be prematurely discontinued due to greater renal toxicity in the combination arm, which was associated with a shorter duration of bacteremia, but no differences in mortality between the groups (145).

The combination of daptomycin and fosfomycin has produced promising results in animal models of MRSA endocarditis (146). This combination has also shown good microbiological results and clinical benefits in patients aged under 70 years in our recent clinical trial of MRSA bacteremia (BACSARM trial) (147), which again had a poor representation of endocarditis cases (diagnosed in 12% of the cases).

Finally, combinations of daptomycin and beta-lactams without and with intrinsic anti-MRSA activity for the treatment of MRSA bacteremia have been shown to be effective in a retrospective study (148) and in a pilot clinical trial (149), respectively, although with little specific information for endocarditis cases.

#### Length of Antibiotic Therapy

Even in infections caused by susceptible microorganisms, the risk of microbiological relapse is plausible in endocarditis. For this reason, long treatments are typically recommended, generally 4 weeks for native valve endocarditis and 6 weeks for PVE. Exceptions to this general rule are the abbreviated 2-week treatments that have proved to be effective for right-sided endocarditis (150) and the combination treatment of beta-lactams and aminoglycosides for native valve endocarditis caused by penicillin-susceptible viridans group streptococci (127). Furthermore, there are clinical trials currently underway that aim to demonstrate the efficacy of shorter-than-standard therapeutic regimens (151).

In native valve endocarditis caused by *E. faecalis*, 4-week aminoglycoside treatment courses are recommended for cases with a clinical evolution shorter than 3 months (152). In cases with a longer symptomatic duration and in those using the double beta-lactam combination, a 6-week treatment course is recommended (153).

The decision of the total duration of antibiotic therapy in patients undergoing surgery during the active phase of treatment is affected by the result of the valve culture (154). According to the findings of a retrospective study, an antibiotic treatment duration of 2 weeks after surgery may be sufficient for valve culture-negative streptococcal endocarditis (155). Management guidelines recommend completing the pre-stipulated duration of the antibiotic treatment course when the valve culture is negative and, conversely, restarting a complete cycle if the valve is not sterile at the time of surgery.

#### Outpatient Parenteral Antibiotic Treatment

Infective endocarditis requires long and expensive hospitalizations for its management given the need to administer parenteral antibiotics for several weeks. Outpatient parenteral antibiotic treatment (OPAT) has been shown to be efficient and cost-effective in the management of endocarditis. A series of specific criteria must be met by the patients for them to be considered safe candidates for OPAT (156). Generally, this option is considered suitable for patients who have overcome the critical phase of the disease (first 2 weeks), provided that they remain stable. Recent studies have demonstrated the safety of this strategy, even expanding the restrictive criteria proposed in the guidelines (157).

#### New Antibiotic Molecules

In recent years, new antibiotics targeting Gram-positive microorganisms (and, therefore, potentially useful for treating endocarditis) have been incorporated. The fifth-generation cephalosporins ceftaroline and ceftobiprole represent the first beta-lactams with intrinsic anti-MRSA activity. Although infective endocarditis is not among the approved indications based on pivotal clinical trials, there are case series that have reported that its off-label use is an effective salvage treatment in patients with endocarditis (158, 159). Ceftaroline and, in particular, ceftobiprole also have anti-enterococcal activity (160), which makes them attractive as candidates for combination treatments with ampicillin or daptomycin in managing enterococcal infective endocarditis (161).

Another interesting new molecule is the lipoglycopeptide dalbavancin, a bactericidal drug with a chemical structure and antibacterial spectrum similar to those of teicoplanin, but with a very long half-life that allows its administration every week or every 2 weeks. Dalbavancin is a potentially useful option for OPAT in selected patients. *In vitro* studies have demonstrated its potent activity against endocarditis-producing strains (162). Furthermore, recently published series of cases indicate that it shows efficacy as a continuation drug in endocarditis cases of diverse etiology (163, 164).

#### Oral Antibiotic Treatment

The effectiveness of oral treatment for endocarditis has been demonstrated for cases involving right-sided valves (165), as stated in the management guidelines. For left-sided endocarditis, retrospective studies (166), a small clinical trial of endocarditis caused by the viridans group streptococci (167) and a recent randomized clinical trial (168) of endocarditis with diverse etiologies (streptococci, enterococci, and staphylococci) have demonstrated that partial oral regimens are effective in treating selected patients who have overcome the critical phase.

In that recent randomized clinical trial, the POET trial, 1,954 patients were evaluated, of whom 400 were finally selected. After a minimum of 10 days of intravenous antibiotic therapy, the patients were randomized to continue intravenous therapy or to complete treatment with a combination of oral drugs. The clinical endpoint was a composite of all-cause mortality, unplanned cardiac surgery, embolic events, and relapse of bacteremia with the primary pathogen from the time of randomization until 6 months after the completion of antibiotic treatment. This endpoint was found to be similar between the groups. Due to the diverse etiologies included in the trial and the various combinations of oral antibiotics administered, the results of the POET trial cannot be used to make specific therapeutic recommendations. However, this trial has raised the possibility of including oral sequential treatment as a suitable option for treatment completion in selected patients.

#### Antimicrobial Treatment for Fungal and Culture-Negative Endocarditis

Antifungal treatment for *Candida* spp. endocarditis is usually based on the use of drugs with fungicidal activity, either based on regimens with liposomal amphotericin B (or other lipid formulations) with or without flucytosine or on regimens based on echinocandins in high doses. Although the evidence is scarce, based mostly on retrospective experiences and expert consensus, the use of combined treatments and long-term azole suppressive treatments is frequent. A high number of cases will require surgical intervention as part of the management (169). On the other hand, in the rare cases of endocarditis due to *Aspergillus* species (often responsible for culture-negative endocarditis), the most commonly used antifungal is voriconazole and the surgery requirements is also high (74, 125).

The appropriate selection of an empirical antibiotic treatment for patients with culture-negative endocarditis is a difficult task and so expert consultation is recommended. The decision is often conditioned by the clinical presentation of the disease, the presence or absence of prosthetic material and other epidemiological data such as other comorbidities, dental hygiene, alcoholism, contact with animals, etc. Thus, for patients with native valves and subacute presentation, empirical treatment should cover viridans group streptococci, enterococci and HACEK (e.g., ampicillin plus either gentamicin or ceftriaxone) whereas in cases with acute clinical presentation and/or presence of prosthetic valves it seems reasonable to cover *Staphylococcus aureus*, coagulase-negative staphylococci, β-hemolytic streptococci and aerobic gram-negative bacilli (e.g., cloxacillin plus ceftriaxone plus either vancomycin or daptomycin) (170, 171) The details on directed treatment options for specific pathogens are available in the published guidelines (74, 125).

### Surgical Treatment

More than 50% of patients with endocarditis will need valve surgery. Among these, a significant proportion will require it during the active phase (that is, during the initial hospitalization and before the end of antibiotic treatment), which is known as early valve surgery (EVS). Although both the American and European guidelines have the same indications for EVS (Table 2) (74, 125), the latter guideline goes further in terms of recommending the timing of surgery. It suggests emergent surgery (within the first 24–48 h) in the case of refractory heart failure secondary to valve regurgitation, and an urgent surgery (during the first week) in almost all other clinical settings.

**Table 2:** Indications for surgery in left-sided valve endocarditis (74).

**Heart failure**
•Severe acute regurgitation, obstruction or fistula causing refractory pulmonary edema or cardiogenic shock.
**Uncontrolled infection**
• Locally uncontrolled infection (abscess, false aneurysm, fistula, or enlarging vegetation). • Persistent positive blood cultures despite appropriate antibiotic therapy. • Infection caused by fungi or multidrug-resistant organisms or prosthetic valve endocarditis caused by staphylococci or non-HACEK Gram-negative bacteria.
**Prevention of embolism**
• Persistent vegetations >10 mm after one or more embolic episode despite appropriate antibiotic therapy or severe valve stenosis or regurgitation. • Isolated very large vegetations (>30 mm).

This contentious issue of the timing of surgery in the guidelines is partly due to the limited and conflicting evidence published to date that is difficult to interpret. There is only one randomized clinical trial that has demonstrated a clinical benefit in terms of a reduction in embolic events for “very” EVS in young patients (median age of 48 years) with native mitral valve endocarditis and mostly caused by streptococcal bacteria (172). It is unclear to what extent this evidence can be extrapolated to older patients and to endocarditis with other etiologies.

Most of the available evidence consists of findings from retrospective studies that require laborious statistical adjustments in order to mitigate indication and survival biases and draw valid conclusions. According to these studies, the benefit of EVS can only be demonstrated for native valve endocarditis with surgical requirement (173). The benefit is less evident in the case of PVE (only demonstrated in patients who are more likely to require surgery) (174) and could be present in some carefully selected patients with PVE caused by *S. aureus* (175).

It seems reasonable to conclude that the decision to proceed with an emergency or urgent valve surgery during the active phase of endocarditis cannot be recommended systematically or routinely. Instead, this decision should be based on an individualized assessment (176).

### Management of Sepsis in Endocarditis

The management of septic shock in patients with endocarditis does not seem to differ from that recommended for sepsis from other sources (59). In addition to volume expansion, vasoactive drugs in some cases and the early initiation of appropriate bactericidal antibiotic treatment, there is still no clinical evidence about the possible effectiveness of additional immunomodulatory treatments in cases of endocarditis and suspected toxic shock (61). It seems appropriate to point out that early valve surgery may be necessary in some cases as a source control measure (30), such as in cases with uncontrolled infection despite correct antibiotic treatment.

## Conclusions and perspective

It is evident that the management of infective endocarditis requires the close collaboration of a multidisciplinary team that includes experts in critical care for patients with severe sepsis or septic shock. The training of endocarditis teams is recommended by the guidelines, while some studies have demonstrated the beneficial impact of their performance on patient outcomes (177, 178).

**Figure 1:**
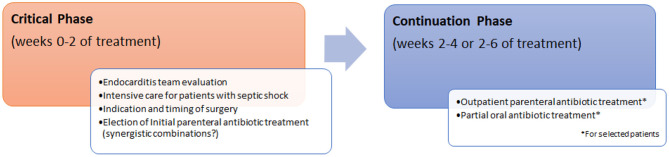
Management of infective endocarditis.

It seems reasonable to conclude that patients with endocarditis should be evaluated preferentially by these multidisciplinary teams in the critical phase of the disease, at which time the most appropriate initial intravenous antibiotic therapy should be chosen. New therapeutics and possible new synergistic combinations of antibiotics are of very high interest. After a thorough clinical and anatomical cardiac evaluation, patients eligible for surgical treatment should be selected and the timing of the intervention decided. It is possible that in coming years, an increasing proportion of appropriately selected patients will be able to continue their antibiotic treatment in home hospitalization regimens with OPAT, using long half-life parenteral antibiotics or combinations of oral antibiotics (Figure 1).

There is a need for international and multi-center working groups to establish a common work agenda in order to scientifically address all the unresolved diagnostic and therapeutic aspects of infective endocarditis.

## Author Contributions

All authors listed have made a substantial, direct and intellectual contribution to the work, and approved it for publication.

## Conflict of Interest

The authors declare that the research was conducted in the absence of any commercial or financial relationships that could be construed as a potential conflict of interest.
